# The impact of high apparent temperature on spontaneous preterm delivery: a case-crossover study

**DOI:** 10.1186/s12940-017-0209-5

**Published:** 2017-02-01

**Authors:** Lyndsay A. Avalos, Hong Chen, De-Kun Li, Rupa Basu

**Affiliations:** 1 Kaiser Permanente Northern California, Division of Research, 2000 Broadway, Oakland, California 94612 USA; 2 California Office of Environmental Health Hazard Assessment, Air and Climate Epidemiology Section, 1515 Clay Street, 16th Floor, Oakland, California 94612 USA

**Keywords:** Spontaneous preterm birth, Heat, Epidemiology, Case-crossover, California

## Abstract

**Background:**

Despite the prediction that temperatures are expected to increase in the future, little is known about the health effects of increasing temperatures on pregnant women. The objective of this study was to investigate the impact of apparent temperature on spontaneous preterm delivery (PTD).

**Methods:**

A case-crossover study of 14,466 singleton spontaneous preterm deliveries occurring between January 1, 1995 and December 31, 2009 among Kaiser Permanente Northern California (KPNC) members was conducted. Preterm deliveries were identified through KPNC's Electronic Health Records (EHR) data. Data on gestational age at delivery, infant sex, and maternal address were also extracted from KPNC's EHR and linked to meteorologic and air pollution monitoring data based on residential zip code.

**Results:**

An 11.6% (95% CI: 4.1, 19.7) increase in spontaneous PTD was associated with a 10 °F (5.6 °C) increase in weekly average (lag06) apparent temperature, during the warm season. During the cold season, increases in apparent temperature did not significantly impact the overall effect of spontaneous PTD (6.2%, (95% CI: -3.0, 16.2) per 10 °F (5.6 °C) increase in weekly average (lag06) apparent temperature). Significant differences in the relationship between apparent temperature and spontaneous PTD emerged for region, gestational age and infant sex, during the cold season. No significant differences emerged for air pollutants.

**Conclusions:**

Our findings provide evidence for an increase in the odds of spontaneous PTD associated with increases in apparent temperature, especially during the warm season.

**Electronic supplementary material:**

The online version of this article (doi:10.1186/s12940-017-0209-5) contains supplementary material, which is available to authorized users.

## Background

Defined as a live birth prior to 37 complete weeks of gestation, preterm delivery (PTD) is the leading cause of perinatal morbidity and mortality, accounting for approximately 30% of early neonatal deaths [1]. In the US and most developed countries, it is the leading cause for congenital neurological disabilities including cerebral palsy, blindness and deafness^1-4^ [2–5], thus signifying the impact it has over the life course of those affected. Each year in the U.S., 12% of live births (~500,000 births) are preterm, resulting in more than $26 billion in medical care costs [6–9]. The combined impact on infant health and extraordinary medical costs make PTD a global health challenge.

The etiology of the majority of preterm births remains unknown. Maternal depression [10], maternal race/ethnicity [11, 12], maternal infections [13, 14], smoking [15, 16] and previous PTD [17] are among several identified risk factors for preterm birth. More recently, research has begun to focus on the impact that environmental factors may have on PTD. Animal models support the biological plausibility of a relationship between extreme heat and adverse pregnancy outcomes including early gestational birth [18–22]. These previous studies give biologic plausibility to the hypothesis that apparent temperature may be a contributing factor for the risk of PTD. However, findings from the limited epidemiological research have been inconsistent [23–32]. Given the expected increase in duration and frequency of heat waves due to climate change and costs of PTD, we investigated the association between maternal exposure to apparent temperature and spontaneous PTD over a 16 year time period (1995 through 2009) in Northern California while taking into account differences by warm and cold seasons, coastal and inland regions, infant sex and gestational age.

## Methods

A case-cross over study was conducted among the Kaiser Permanente Northern California (KPNC) member population of women who delivered a singleton, live preterm delivery between January 1, 1995 and December 31, 2009. KPNC, a large group practice within an integrated health care delivery system, provides comprehensive medical services to over 3.6 million members and has approximately 37,000 deliveries in a 14-county region per year. Coverage is provided for approximately 30% of the northern California population and is similar demographically, racially and ethnically to the population living in the geographic area [33, 34]. KPNC has well established automated clinical databases that capture delivery dates and gestational age at delivery.

The time-stratified case-crossover approach has been utilized in previous research assessing various acute outcomes associated with meteorologic exposures and air pollution [26, 35, 36]. Briefly, in the case-crossover method each person serves as his/her own control, thus time in-variant factors both known (e.g., race/ethnicity) and unknown are adjusted for by design [37]. Apparent temperature for the seven days prior to the infant’s birth was considered the exposure period for the mother. The control periods for each mother consisted of seven day intervals during the same month and year of the infant’s birth and were limited to the same day of the week as the birth. Each mother could have up to a maximum of 4 control periods.

Meteorologic and air pollution data were ascertained through linkage to various databases including the California Irrigation Management Information System [38], the US Environmental Protection Agency Air Quality System [39], and the California Air Resources Board [40]. To examine the relationship between apparent temperature and PTD, we included all live, non-induced preterm deliveries. The study was approved by the Kaiser Permanente Northern California Internal Review Board.

### Ascertainment of PTD

**Fig. 1:**
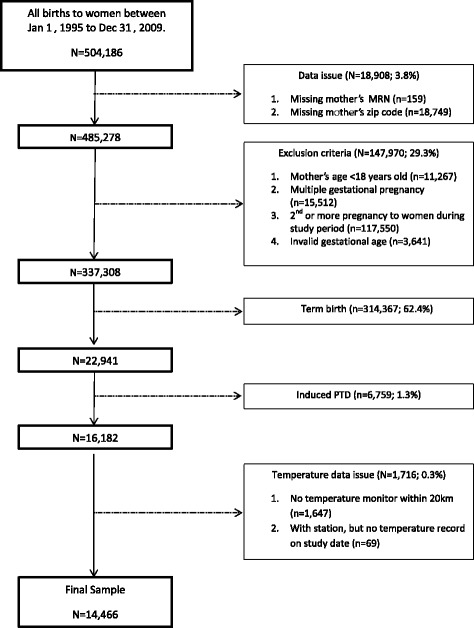
Flowchart of Study Exclusion and Inclusion Criteria

Gestational age at delivery was ascertained from clinical databases and based on best clinical estimate, first day of last menstrual period (LMP) and ultrasound. In more recent years gestational age is primarily based on ultrasound. Live births to women <18 years of age, whose zip code was not available in the EHR, who did not have a valid gestational date (e.g., <16 weeks, >45 weeks, or missing (<1% of live births)), or who had a multiple gestation were excluded. Additionally, only a woman’s first birth during this time period was included to avoid non-independent observations. PTD was defined as a live birth prior to 37 complete weeks of gestation. Deliveries induced preterm because of pregnancy complications were excluded as defined by the following ICD9 codes (73.0, 73.01, 73.09, 73.1x, 73.4x, and 74.x (without codes indicating labor or spontaneous delivery)) [41]. A flowchart outlining the inclusion/exclusion criteria is included in Fig. 1.

### Ascertainment of apparent temperature

Meteorological data consisting of daily mean, maximum and minimum temperatures and relative humidity and dew point temperatures were ascertained and used to calculate apparent temperature with the following formula [42]: ‐ 2.653 + (0.994 × temperature in ° C) + 0.0153 × (dew ‐ point temperature in ° C)^2^. Each mother was assigned the resulting apparent temperature value from the weather monitor closest to the reported residential zip code centroid at the time she gave birth. Cases residing in zip codes with centroids located outside 20 km of a weather monitor were ineligible for this study (7%). Please see Additional file 1: Figure S1 for a map of the temperature monitors in the study area.

### Ascertainment of potential confounders and effect modifiers

Maximum daily data from air pollution monitors were provided by the California Air Resources Board [43]. The pollution monitor closest to each mother's residential zip code centroid was used to assign exposure to carbon monoxide (CO), nitrogen dioxide (NO_2_), sulfur dioxide (SO_2_), and ozone (O_3_), restricting cases to those residing within 10 km for analyses. For particulate matter (PM_2.5_) the mean daily data were provided.

Effect modifiers considered were region (coastal versus inland regions), gestational age (severe preterm (<35 weeks), moderate preterm (35-36 weeks), and near term (>36, <37 weeks)) and infant sex (male, female).

### Statistical analysis

Conditional logistic regression was conducted using a linear term for apparent temperature and preterm birth as the outcome separately for each county based on residential zip code. A random-effects meta-analysis was performed to calculate an overall estimate [44]. All estimates are reported as percent change per 10 °F (5.6 °C) increase in apparent temperature with corresponding 95% confidence intervals. Given that apparent temperature is not linear year round but is linear during warm and cold seasons, all analyses were stratified by warm season (deliveries between May 1 and October 31) and cold season (deliveries between November 1 and April 31). Counties with less than 5 PTD cases after stratification were excluded from the respective analysis. Conditional logistic regression in SAS version 9.3 was used to conduct the first stage of the analysis, and STATA version 10.1 was used for the meta-analyses.

The mean, maximum and minimum apparent temperature for 6-single day lags (lag 1, lag 2, lag 3, lag 4, lag 5, and lag 6), and 3 cumulative average lag periods (lag01, lag03, lag06) were examined to determine the best fit model. The best fit model criterion was based on a combination of the -2 logL statistic (deviance) and potential biological underlying mechanisms for the relationship between temperature and PTD. The single-day lags represent apparent temperature on the previous day(s) (e.g., lag 1 refers to the apparent temperature on the previous day, lag 2 refers to the apparent temperature 2 days prior, etc). The cumulative lag periods represent averages of several days (e.g., lag06 represents the exposure on the same day and the previous 6 days).

To assess confounding by air pollutants, the maximum lag01 for each of the air pollutants (mean lag01 for PM_2.5)_was included in a separate model with apparent temperature and PTD and compared to the unadjusted model. Potential effect modification by air pollutants was assessed by including an interaction term between apparent temperature and the maximum lag01 of each air pollutant (mean lag01 for PM_2.5_), in five distinct models. As tests for interaction generally have less power to test for statistical significance [45], a priori a *p*-value < 0.10 was considered as statistically significant.

We used the best fit model to examine other potential effect modifiers of high apparent temperature and PTD including region (coastal versus inland), and gestational age at delivery (severe preterm (<35 weeks), moderate preterm (35-36 weeks), and near term (>36, <37 weeks) deliveries), and by infant sex. All analyses were conducted separately for the cold and warm season.

### Post hoc analysis

Given evidence suggesting an increase in health risk for early term gestations, we conducted additional analyses assessing the relationship between apparent temperature and births between 37-38 weeks gestation.

### Sensitivity analysis

A sensitivity analysis was conducted to address the potential of fixed cohort bias impacting our findings [46]. The association between increasing apparent temperature and PTD was assessed for both the warm and cold season restricting the sample to women with conception dates between August 28, 1994 (19 weeks before the cohort started) and March 5, 2009 (43 weeks before the cohort ended).

## Results

The study population included 14,466 spontaneous preterm births (6,497 during the warm season and 7,969 during the cold season) and their characteristics are shown in Table 1. A majority of the women in the study were between 25- 34 years of age (54%), or White (40%). For the warm season, the mean, 5^th^ percentile and 95^th^ percentile values for the case mean, minimum, and maximum apparent temperature, as well as maximum ozone, carbon monoxide, nitrogen dioxide, sulfur dioxide and mean particulate matter PM_2.5_ are listed by county in Table 2 ((a) overall mean: 64.5 °F(1 °F = 0.56 °C), (b) case minimum apparent temperature 53.4 °F, (c) case maximum apparent temperature 77.5 °F, (d) ozone (47.5 ppb), (e) carbon monoxide (0.9 ppm), (f) nitrogen dioxide (25.2 ppb), (g) sulfur dioxide (3.6 ppb) and (h) PM_2.5_ (10.6 µg/m^3^)). Table 3 lists the mean, 5^th^ percentile and 95^th^ percentile values for the case mean, minimum, and maximum apparent temperature, as well as maximum ozone, carbon monoxide, nitrogen dioxide, sulfur dioxide and mean particulate matter PM_2.5_ by county for the cold season ((a) overall mean: 49.2 °F (26 °C), (b) case minimum apparent temperature 40.7 °F, (c) case maximum apparent temperature 58.5 °F, (d) ozone (33.5 ppb), (e) carbon monoxide (1.6 ppm), (f) nitrogen dioxide (31.9 ppb), (g) sulfur dioxide (3.7 ppb), and (h) PM_2.5_ (16.2 µg/m^3^)).

**Table 1:** Characteristics of the Study Population, by California County, 1995-2009 (*N* = 14,466)

County	N^a^	Maternal Age (years), %				Maternal Race/Ethnicity, %					Infant Sex,%
		18-19	20-24	25-34	>35	White	Black	Hispanic	Asian	Other^b^	Male
Alameda	2,362	5	17	55	24	27	14	22	26	12	54
Butte^c^	24	21	29	38	13	71	0	13	13	4	58
Contra Costa	1,467	5	16	54	24	45	11	18	16	10	55
El Dorado/Amador	95	5	6	66	22	82	0	7	6	4	60
Madera	39	10	21	41	28	38	5	51	3	3	59
Marin	198	2	13	58	27	60	5	21	10	5	51
Merced	17	6	24	35	35	47	0	29	0	24	59
Monterey	17	0	35	41	24	35	0	47	12	6	59
Napa/Lake	166	6	19	54	20	62	0	33	2	2	58
Nevada/Fresno	476	8	22	53	17	43	7	38	9	3	55
Placer	311	5	18	59	18	70	2	13	11	4	50
Sacramento	2,489	8	21	51	20	49	14	17	17	4	56
San Benito	24	4	8	54	33	50	8	38	0	4	63
San Francisco	800	3	13	54	30	30	12	19	34	6	59
San Joaquin	661	7	17	57	19	31	10	33	14	12	55
San Mateo	878	4	15	55	26	30	2	28	34	6	56
Santa Clara	2,441	4	16	57	23	35	3	29	24	9	54
Santa Cruz	18	6	17	33	44	72	0	6	0	22	61
Solano	1,018	8	23	50	19	40	19	16	20	5	54
Sonoma/Mendocino	566	6	17	56	20	67	1	23	6	2	57
Stanislaus	211	3	19	62	16	33	3	30	6	28	48
Sutter	13	8	15	54	23	46	15	23	8	8	62
Tulare/King	17	6	41	35	18	24	0	65	0	12	59
Yolo	158	6	20	56	18	53	3	27	16	1	51
Total	14,466	6	18	54	22	40	9	23	20	8	55

^a^N: number of preterm births;

^b^Other: American Indian, other/unknown race

^c^5 Combined counties includes: Butte, Lassen, Shasta, Tehama, Yuba

**Table 2:** Exposure Metrics for Weekly Average (Lag06) Apparent Temperature and Air Pollutants during the Warm Season, by California County, 1995-2009 (*n* = 6497)

County	Case Mean Apparent Temperature, °F (5^th^, 95^th^)	Case-Control Difference °F	Minimum Apparent Temperature, °F (5^th^, 95^th^)	Maximum Apparent Temperature, °F (5^th^, 95^th^)	Max O_3_^a^, ppb (5^th^ ,95^th^ )	Max CO^a^, ppm (5^th^,95^th^)	Max NO_2_^a^, ppb (5^th^,95^th^ )	Max SO_2_^a^, ppb (5^th^,95^th^ )	Mean PM_2.5_^a^, µg/m^3^ (5^th^ ,95^th^ )
Alameda	61.7(54.0-70.4)	3	52.9(44.9-59.8)	72.6(61.7-85.1)	39.3(21.5-68.5)	0.9(0.3-2.2)	26.2(9.5-51)	3.3(0.5-8.5)	12.8(5.7-24.1)
Butte^b^	67.2(49.7-76.5)	6	52(31.9-62.3)	82.7(65.5-92.1)	57.8(37.5-85.5)	0.6(0.4-0.9)	26.6(22-36)	N.A.	15.4(2.8-81.3)
Contra Costa	64.6(54.3-75.1)	3.6	52.5(43.3-60.2)	78.7(64.6-92.8)	47.3(27.0-79.5)	0.7(0.3-1.8)	22(9-42.5)	5.0(1.0-13.5)	8.2(4.1-14.4)
El Dorado/Amador	67.6(51.5-83.0)	5.7	54.6(40.6-65.9)	81(60.1-99.3)	70.7(44.0-108.0)	0.5(0.2-1)	N.A.	N.A.	N.A.
Madera	70.7(54.7-95.5)	4.5	54.9(39.9-80.8)	86.3(70.4-109.9)	N.A.	N.A.	N.A.	N.A.	N.A.
Marin	59.1(51.8-66.1)	3.2	47.6(39.4-54.4)	73.1(60.5-87.4)	32.9(19.5-51.0)	0.9(0.4-2.2)	21.1(11-36.5)	N.A.	N.A.
Merced	65.1(50.8-74.3)	5.6	48.4(36.4-56.7)	81.5(63.9-94.7)	64.3(43.0-97.0)	N.A.	N.A.	N.A.	N.A.
Monterey	57.5(52.8-63.7)	1.3	50.6(45.5-55.9)	66.3(59.7-77.3)	36.0(24.5-52.5)	0.9(0.5-1.6)	15.2(6-32.5)	N.A.	N.A.
Napa/Lake	60.9(52.1-68.7)	3	46.4(38.5-54.1)	77(63.6-88.8)	43.3(28.5-65.0)	0.8(0.2-2.1)	18.5(7.5-37.5)	N.A.	N.A.
Nevada/Fresno	73.8(60.8-86.0)	4.4	59.7(48.2-73.1)	87(70.9-99.6)	76.4(45.5-113.0)	0.8(0.2-1.9)	30.4(13-58.5)	4.4(0.5-11.0)	12.1(5.0-27.5)
Placer	69.4(56.1-80.9)	4	56.9(46.4-66.2)	82.6(66.7-95.8)	63.5(37.5-96.5)	0.8(0.3-1.6)	31.6(9.5-61)	2.4(0.5-5.5)	N.A.
Sacramento	68.9(56.9-80.2)	4.3	55.6(45.4-64.9)	83.7(68.3-96.4)	61.5(37.0-92.5)	0.9(0.3-2.3)	25.2(8.5-51.5)	3.4(0.5-9.0)	9.6(4.0-17.5)
San Benito	58.7(48.6-69.2)	3	46.9(38.4-58.8)	72.5(59.1-82.5)	48.3(34.0-71.5)	N.A.	N.A.	N.A.	N.A.
San Francisco	59.6(53.0-66.1)	2.5	53(47.2-58.6)	68.8(59.4-77.7)	32.3(21.0-45.5)	1.1(0.4-2.4)	24.9(10-51.5)	3.0(0.5-9.0)	11.2(4.8-19.9)
San Joaquin	68.6(56.1-78.8)	4.1	55(43.7-65.4)	83.2(68.1-94.8)	56.0(34.0-83.0)	0.9(0.3-2.1)	30.2(15.3-54.5)	N.A.	17.9(9.0-52.0)
San Mateo	59.9(53.3-67.3)	2.7	53.1(47.3-59.7)	68.9(60.7-78.4)	34.1(22.0-51.0)	1(0.4-2.4)	24.1(11.5-42.5)	2.9(0.0-8.5)	10.6(5.0-19.2)
Santa Clara	64.0(55.4-71.8)	3.3	54.2(46.1-61.5)	75.9(64.7-85.7)	43.6(26.5-72.0)	1.2(0.3-3)	32.4(14.5-59.5)	0.7(0.2-1.6)	11.5(5.3-22.3)
Santa Cruz	57.8(52.0-63.4)	1.9	48(43.2-53.5)	69.8(58.7-79.1)	42.1(27.0-64.5	N.A.	N.A.	N.A.	N.A.
Solano	63.5(53.8-73.5)	3.3	51.2(41.6-58.8)	78.1(64.9-91)	44.7(27.0-73.5)	0.8(0.3-2.8)	18.8(7-39)	3.2(1.0-8.5)	8.8(3.8-14.2)
Sonoma/Mendocino	58.5(48.9-66.0)	3.1	43.9(34-51)	76.1(61.7-86.9)	37.7(24.5-55.5)	0.7(0.3-1.7)	20.3(8-37.5)	N.A.	5.8(3.7-7.9)
Stanislaus	69.9(59.1-82.7)	4.2	56(45.7-69)	84.7(70.4-97.7)	61.3(36.5-91.5)	0.7(0.1-2.4)	30.7(9.5-66.5)	N.A.	6.0(6.0-6.0)
Sutter	66.2(55.5-75.9)	3.2	52.5(42.7-61)	80.2(69.5-90.1)	50.6(23.5-77.5)	1.2(0.2-3.3)	31.7(12-56)	N.A.	6.6(3.5-8.5)
Tulare/King	71.9(63.6-81.3)	2.4	56.8(48.4-66.4)	85.7(76.9-96.4)	81.1(48.0-113.0)	0.8(0.5-1.1)	25.7(10.5-53.5)	N.A.	12.0(12.0-12.0)
Yolo	68.8(56.3-79.9)	4	54(43.5-63.9)	84.7(67.3-97.8)	58.4(38.5-85.0)	0.6(0.2-1.9)	23.6(10.5-47)	N.A.	9.5(4.0-15.5)
Total	64.5(54.2-77.1)	3.5	53.4(43.5-62.8)	77.5(63.2-93.3)	47.5(25-84)	0.9(0.3-2.3)	25.2(9-51)	3.6(0.5-10)	10.6(4.5-20.5)

*Abbreviations*: *CO* carbon monoxide, *NO*_*2*_ nitrogen dioxide, *O*_*3*_ ozone, *SO*_*2*_ sulfur dioxide, *PM*_*2.5*_ particulate matter less than 2.5 lm in aerodynamic diameter

^a^﻿﻿Air pollution data restricted to monitors within 10 km of cases

^b^5 Combined counties includes: Butte, Lassen, Shasta, Tehama, Yuba

**Table 3:** Exposure Metrics for Weekly Average (Lag06) Apparent Temperature and Air Pollutants during the Cold Season, by California County, 1995-2009 (*n* = 7969)

County	Case Mean Apparent Temperature, °F (5^th^, 95^th^)	Case-Control Difference °F	Minimum Apparent Temperature, °F (5^th^, 95^th^)	Maximum Apparent Temperature, °F (5^th^, 95^th^)	Max O_3_^a^, ppb (5^th^,95^th^ )	Max CO^a^, ppm (5^th^,95^th^)	Max NO_2_^a^, ppb (5^th^,95^th^ )	Max SO_2_^a^, ppb (5^th^,95^th^)	Mean PM_2.5_^a^, µg/m^3^ (5th, 95^th^)
Alameda	49.6(41.8-57.2)	3.3	41.4(31.9-50.6)	58.4(49.8-68.2)	33.3(16.5-50)	1.5(0.5-3.2)	32.9(18.5-48.5)	4.3(1-8.5)	11.7(3.3-30.9)
Butte^b^	48.2(38.1-55.3)	3.6	38.9(30.1-47.8)	58.1(47.1-68.7)	27.6(14-41.5)	3(0.9-5.3)	32.8(19-43.5)	N.A.	12.3(3.4-23.5)
Contra Costa	48.6(39.6-57.9)	3.7	40(30.5-49)	58.3(47.8-71.6)	34.8(15.5-52.5)	1.3(0.4-2.8)	28.5(14.5-42)	4(1-10)	13(3.5-35.8)
El Dorado/Amador	45.8(37.8-56.2)	3.9	37.9(30.1-46.4)	54.9(44.3-67.8)	39.5(22.5-62.5)	0.5(0.2-0.8)	N.A.	N.A.	N.A.
Madera	47.2(37.9-61.3)	4.3	36.6(26.8-45.8)	58.5(48-76.6)	N.A.	N.A.	N.A.	N.A.	N.A.
Marin	46(38.2-53.6)	3.1	37.9(28.6-46)	55(46.5-65)	28.1(10.5-43)	1.5(0.6-3.5)	30.5(18.5-45)	N.A.	N.A.
Merced	46(39.5-52.8)	2.1	36.2(31.3-43.5)	57.1(49.5-66.7)	36.1(29-45.5)	N.A.	18.9(9.5-30.5)	N.A.	N.A.
Monterey	45.8(34.7-53.1)	3.9	37.3(24.4-47.4)	55.9(50.4-63.1)	37.3(22.5-49.5)	1(0.3-1.8)	19.8(6-32)	N.A.	N.A.
Napa/Lake	47.2(38.2-55.2)	4.1	36.7(26.6-47)	58.2(49.7-69.4)	32.9(16.5-48)	1.6(0.5-3.1)	26.6(13-38.5)	N.A.	N.A.
Nevada/Fresno	49.4(39-61.6)	4.5	39.9(30.2-49.3)	59.3(47.2-74.8)	42(16.5-72.5)	1.6(0.4-5)	35.5(19-58.5)	2.4(0-7)	28(5.5-66.5)
Placer	48.6(37.5-60.1)	4.3	40.4(30.4-49.7)	57.5(45.3-70.6)	34.1(15.5-54.5)	1.1(0.4-2.4)	28.8(8.5-45.5)	1.8(0-5)	N.A.
Sacramento	48.9(38.9-59.8)	4.1	40.3(30.4-49.5)	58.4(46.6-73.2)	36(14-58.5)	1.6(0.5-3.8)	30(14-46.5)	3.3(0-10)	16.9(4-48)
San Benito	51.6(47.7-57.1)	4	41.3(36.9-52.4)	63.2(56.6-70.2)	42(28.5-53)	N.A.	N.A.	N.A.	N.A.
San Francisco	50.9(44.4-57.4)	3	45(38.2-51.8)	57.6(50.3-66.7)	30(13-45)	1.6(0.6-3.3)	37(22.5-52.5)	4.6(1-10.5)	13(4.3-30.3)
San Joaquin	48.4(38.1-60.1)	4.1	39(28.9-48)	58.8(47.1-73.3)	31.6(9.5-55)	1.6(0.5-4.1)	33.1(22-48.5)	N.A.	22.9(5.5-45.5)
San Mateo	50(43.8-56.5)	2.8	43.7(37.3-50.2)	57(49.5-65.4)	31.1(13-46.5)	1.9(0.7-4)	31.8(18.5-46)	4.4(1-10)	13.1(4.2-30.1)
Santa Clara	50.6(42.3-59.3)	3.5	42.2(33.6-50.5)	59.9(50.9-70.7)	30.9(12-48.5)	2.2(0.6-5.3)	39.7(24.5-61)	1.3(0.1-3.2)	15.5(4.4-40.8)
Santa Cruz	44.4(38.2-48.8)	4.6	35.3(28.4-40.8)	54.3(48.2-58.7)	35.1(23.5-45)	N.A.	N.A.	N.A.	N.A.
Solano	48.7(39.3-58.8)	3.7	39(29.3-47.6)	58.8(48.4-72.1)	34(16-51)	1.8(0.4-4.1)	28.5(14-41.5)	3.6(1-8)	14(3.3-36.8)
Sonoma/Mendocino	45.7(36.5-54.8)	3.8	35.2(24.8-46.3)	58(47.5-70.5)	32.5(11.5-46.3)	1.3(0.5-2.8)	27.4(16.5-37.5)	N.A.	14.5(3.1-33.5)
Stanislaus	47.4(38.2-57.8)	3.6	37.5(26.4-45.7)	58.2(48-70.7)	33.7(12-59)	1.2(0.4-3)	29.2(16.5-45.5)	N.A.	7(7-7)
Sutter	50.7(41.1-58.6)	5.1	36.9(25.3-44.9)	66.6(61.6-71.3)	42.1(20-64)	1.6(0.4-4)	34.6(23.5-43.5)	N.A.	N.A.
Tulare/King	41(34.8-48)	5.4	32.4(25.6-41.4)	50.6(44.4-55.2)	26.3(12-40.5)	1.6(0.6-3.3)	30.3(19.5-38)	N.A.	N.A.
Yolo	47.6(39-55.9)	3.4	38.5(30.8-46.7)	57.5(45.5-68.4)	33.5(14-56)	1.2(0.4-2.9)	29.7(15.5-49)	N.A.	16.5(3.5-41)
Total	49.2(39.7-58.4)	3.6	40.7(30.6-49.8)	58.5(48.2-70.7)	33.5(14-53.5)	1.6(0.5-3.6)	31.9(16-49)	3.7(0.5-9.5)	16.2(4-43.2)

*Abbreviations*: *CO* carbon monoxide, *NO*_*2*_, nitrogen dioxide; *O*_*3*_, ozone; *SO*_*2*_, sulfur dioxide; *PM*_*2.5*_, particulate matter less than 2.5 lm in aerodynamic diameter

^a^Air pollutant data restricted to monitors within 10 km of the cases

^b^5 Combined counties includes: Butte, Lassen, Shasta, Tehama, Yuba

**Fig. 2:**
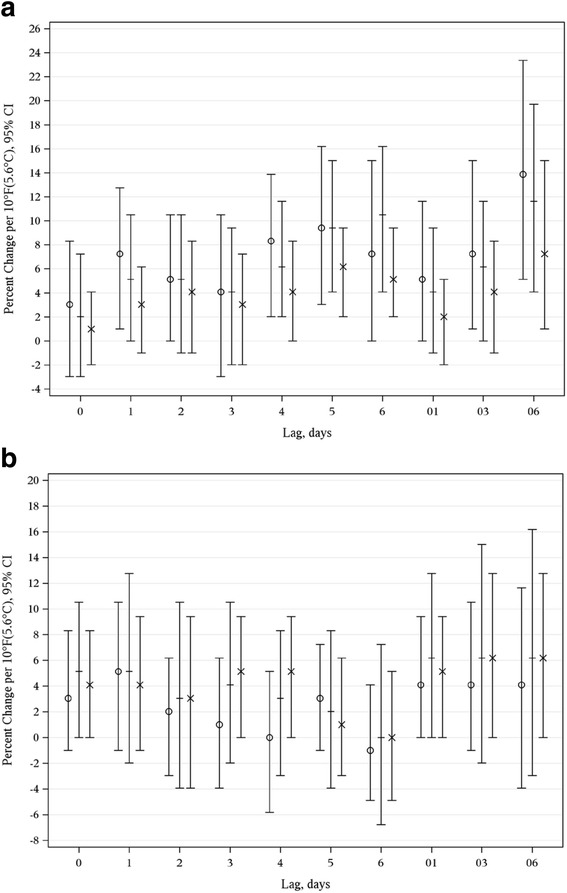
**a** Estimated Percent Change Associated with a 10 °F(5.6 °C) Increase in Mean, Minimum, and Maximum Apparent Temperature and Spontaneous Preterm Delivery by Lag Time during the Warm Season. **b** Estimated Percent Change Associated with a 10 °F(5.6 °C) Increase in Mean, Minimum, and Maximum Apparent Temperature and Spontaneous Preterm Delivery by Lag Time during the Cold Season. O Minimum + Mean X Maximum

The cumulative average weekly lag (lag06) provided the best fit model for the mean, maximum and minimum apparent temperatures (Figs. 2a and b) for both the warm and cold seasons. However, during the warm season an elevated odds of PTD was also associated with other temperature metrics occurring on 4, 5 and 6 days prior to delivery. Given the mean apparent temperature provided the best fit model across lags, the average weekly lag06 for the mean apparent temperature was chosen for the following analyses by warm and cold season.

**Fig. 3:**
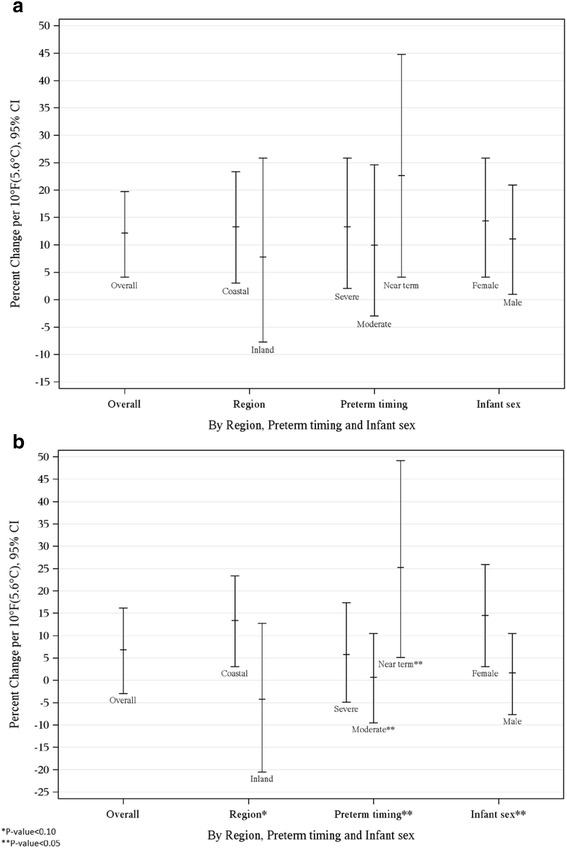
**a** Estimated Percent Change Associated with a 10 °F(5.6 °C) Increase in Weekly Average Apparent Temperature and Spontaneous Preterm Delivery during the Warm Season, Overall and by Region, Preterm Delivery Timing and Infant Sex. **b** Estimated Percent Change Associated with a 10 °F(5.6 °C) Increase in Weekly Average Apparent Temperature and Spontaneous Preterm Delivery during the Cold Season, Overall and by Region, Preterm Delivery Timing and Infant Sex

During the warm season, an 11.6% (95% CI: 4.1%, 19.7%) increase in spontaneous PTD was associated with a 10 °F (5.6 °C) increase in weekly average (lag06) mean apparent temperature (Fig. 3a). An elevated odds of PTD was noted for the coastal region (12.75%, 95%CI: 3.05%, 23.37%) with a trend emerging in inland regions (7.25%, 95%CI: -7.69%, 25.86%). Differences between the estimates for coastal versus inland region were non-significant (*p* = 0.59). A 22.1% increase in the odds of PTD was found for near term PTD (95% CI: 4.1%, 44.8%) and a 12.8% increase was noted for severe preterm deliveries. A non-significant increase in the odds of PTD was found for moderate preterm deliveries (9.4%, 95% CI: -2.0%, 24.6%). Differences between the timing of delivery were not significant (*p* =0.31 between near term and moderate preterm deliveries). Female infants had a slightly more elevated odds (13.9%, 95% CI: 4.1%, 25.9%) but they were not significantly different from that of male infants (10.5%, 95% CI: 1.01%, 20.9%; *p* = 0.66).

A non-significant elevated odds of spontaneous PTD was found during the cold season (6.2%, (95% CI: -3.0%, 16.2%) per 10 °F (5.6 °C) increase in weekly average (lag06) mean apparent temperature) (Fig. 3b). A differential odds of PTD was found by region (*p* = 0.08). For the coastal region, a 12.8% (95% CI: 3.1%, 23.4%) increase in PTD emerged, however a slightly non-significant decreased odds was noted for the inland region (-4.9%, 95%CI: -20.5%, 12.75%). The strongest impact of mean apparent temperature on the gestational age of delivery was observed for near term deliveries (24.6%, 95% CI: 5.1%, 49.2%) which was significantly different from moderate preterm deliveries (0.00%, 95%CI: -9.5%, 10.52%; *p* = 0.04), although not statistically different from severe preterm deliveries (5.1%, 95% CI: -4.9%, 17.4%, *p* = 0.11). A significant elevated odds was found for female infants (13.88%, 95% CI: 3.05%, 25.9%), whereas male infants had a slightly elevated, but non-significant increased odds (1.01%, 95% CI: -7.7%, 10.5%). Differences between the estimates by infant sex were significant (*p* = 0.01).

**Fig. 4:**
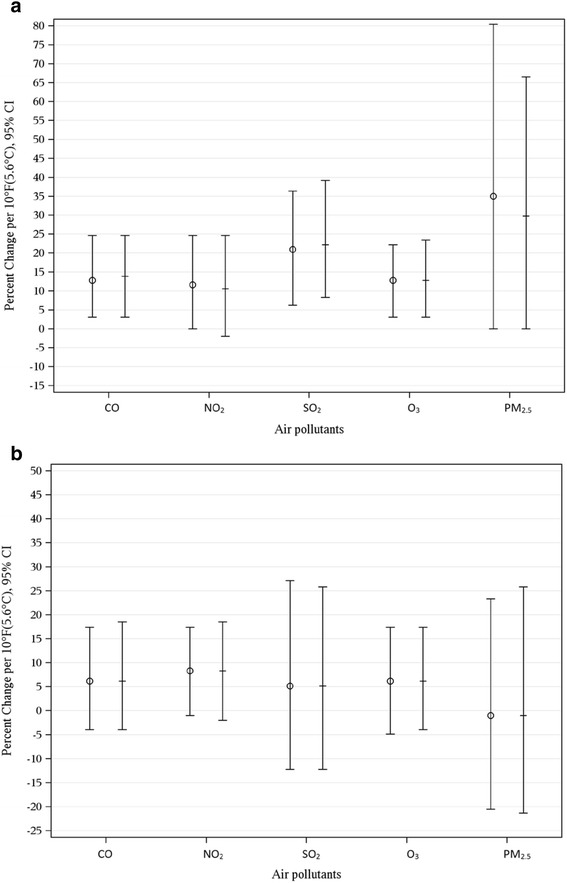
**a** Crude and Adjusted Models Estimating the Percent Change Associated with a 10 °F(5.6 °C) Increase in Weekly Average Apparent Temperature and Spontaneous Preterm Delivery for Five Air Pollutants (CO, NO_2_, SO_2_, O_3_, and PM_2.5_) during the Warm Season. **b** Crude and Adjusted Models Estimating the Percent Change Associated with a 10 °F(5.6 °C) Increase in Weekly Average Apparent Temperature and Spontaneous Preterm Delivery for Five Air Pollutants (CO, NO_2_, SO_2_, O_3_, and PM_2.5_) during the Cold season. O Crude + Adjusted

Air pollutant data was available on a subset of women ranging from 8% (PM_2.5_) to 80% (ozone) during the warm season and 17.3% (PM_2.5_) to 74% (ozone) during the cold season. No significant confounding was found for air pollutant data in either the warm or cold season (Figs. 4a and b). There was no significant interaction between mean apparent temperature and any air pollutant on the odds of PTD for either the warm or cold season.

A non-significant 4.1% (95% CI: -1%, 6.2%) increase in the odds of spontaneous early term births was associated with a 10 °F (5.6 °C) increase in weekly average (lag06) mean apparent temperature, during the warm season. During the cold season, there was no association between an increase in weekly average (lag06) mean apparent temperature and early term births (0% (95% CI: -3.9%, 5.1%)).

Our sensitivity analysis indicated an 11.6% (95% CI: 3.0%, 22.1%) increase in spontaneous PTD associated with a 10 °F (5.6 °C) increase in weekly average (lag06) mean apparent temperature during the warm season. During the cold season, a non-significant increase emerged (6.2% (95% CI: -3.0%, 17.4%)).

## Discussion

We observed an increase in the odds of spontaneous PTD associated with escalations in apparent temperature, especially during the warm season. Our sensitivity analyses replicated these results, further supporting our findings. An increase in the mean, minimum and maximum apparent temperature over the six days prior to delivery (lag06) were all significantly associated with an increase in PTD. However, an elevated odds of PTD associated with other temperature metrics was also noted specifically those for 4, 5 and 6 days prior to delivery, as opposed to more proximal days to delivery. During the cold season, increases in apparent temperature did not significantly impact the overall effect of PTD. Our findings suggest that during the warm season, it may take exposure to a week of increases in the average apparent temperature to impact spontaneous PTD.

The differences highlighted between seasons in our study may help explain the ambiguity of the relationship between increases in apparent temperature and PTD that has emerged in the literature. With one exception [47], studies conducted in more temperate climates such as Montreal, London and Germany [25, 29, 48] failed to find a heat-related effect on preterm deliveries. Yet, a majority of the research conducted in warmer climate regions has noted an increase in preterm labor and/or deliveries associated with increases in apparent temperature [23, 26–28, 30, 31]. Only one study in warmer climate failed to find a relationship [32]. It is possible that there is a minimum threshold at which heat triggers a labor response resulting in PTD, yet more research needs to be done to confirm this hypothesis.

Women living in coastal regions were impacted more by increases in apparent temperatures, especially during the cold season. Although a significant differential effect did not occur during the warm season, we noted a similar trend. This is of interest given the higher temperatures that women are exposed to inland compared to in coastal regions. In Northern California, homes in the coastal region are less likely to have air conditioning [49]. Thus one possible explanation for these findings is that women living in these areas may have fewer opportunities to protect themselves from increases in heat. Another explanation could be biological acclimatization as women who live in inland regions are used to hotter temperatures.

Increases in temperature appeared to have the strongest impact on severe and near term preterm deliveries, especially during the cold season. We acknowledge that small cell sizes increase the possibility that these findings may be due to chance. Few studies have evaluated the timing of the PTD and the findings have not been consistent [23, 26, 28]. While two studies [24, 26] found preterm births occurring later in pregnancy had greater associations than those occurring earlier, an additional study [23] found stronger associations for preterm deliveries occurring earlier. The final study that found an association with later PTD assessed all deliveries between 28 and 37 weeks gestation together [28]. Additionally, although inconsistent with another study, our posthoc analyses did not appear to support a relationship between increasing temperatures and early term births [26].

Finally, similar to several other studies [23, 26, 28, 47] we found an association between apparent air temperature and PTD independent of air pollutants.

### Biological mechanism

A clear biological mechanism underlying escalations in temperature and preterm birth is currently unknown. Animal models have documented an increase in two hormones as a result of heat exposure stress (prostaglandin F_2α_ (PGF_2α_) and oxytocin) [19–21], which are also associated with labor induction in humans [50–54]. Heat related stress also leads to an increase in heat-shock proteins (HSP) [55] which have been linked to PTD [56, 57]. The effects of HSP on PTD may be due to pro-inflammatory cytokines impacting prostaglandin E2 secretion which is known to be involved in initiation of parturition [58–60]. Additionally, dehydration caused by heat exposure can constrict uterine blood flow, and increase pituitary secretion of antidiuretic hormones and the labor inducing hormone, oxytocin [61]. Heat stress may exacerbate the potential inefficient thermoregulation by pregnant women and may increase the amount of blood shifted from vital organs of the mother and fetus [62]. High temperatures may also increase blood viscosity and cholesterol levels which may impact labor induction [63]. Findings from our study are consistent with the mechanisms outlined above regarding the immediate effects of heat exposure on PTD.

### Limitations

We note a few limitations of the study. Ecological level data was used to assess temperature as individual heat monitoring data was not available. However, we minimized the potential for misclassification by limiting the study to pregnant women residing in residential zip codes within 20 kilometers of a meteorological monitor. This criteria also addresses potential exposure misclassification due to residential mobility as a majority of women who move during pregnancy tend to stay within the same exposure region [64]. Additionally, we lacked information on residential or occupational air conditioning status. However, a majority of homes in the coastal region in Northern California do not have air conditioning [49]. Since most homes on the coast are more expensive, the lack of residential air conditioning is not a marker of socioeconomic status or bias.

Spanning 16 years, this study contributes one of the longest periods of time used to assess the relationship between apparent temperature and PTD and confirms previous research suggesting an increase in the odds of spontaneous PTD associated with increases in apparent temperatures, especially during the warm season. The use of electronic health records provide a more accurate method to differentiate spontaneous versus induced preterm deliveries and decrease misclassification of spontaneous PTD. The findings from the inclusion of both the warm and cold season in this study provide some indication that there may be a threshold at which apparent temperature triggers a labor response resulting in spontaneous PTD. Given that heat waves are likely to increase in severity and duration in the future^1^, the findings from this study have significant public health implications for pregnant women.

## Conclusions

Our findings suggest an increase in the odds of PTD associated with increases in ambient temperatures, especially during the warm season. Given that heat waves are likely to increase in severity and duration in the future^1^, the findings from this study have significant public health implications for pregnant women.

## Additional file

Additional file 1: Figure S1. Map of Temperature Monitors in Northern California Counties Represented in the Study. (PDF 2141 kb)

## Abbreviations

CO: Carbon monoxide
EHR: Electronic health records
KPNC: Kaiser Permanente Northern California
NO_2_: Nitrogen dioxide
O_3_: Ozone
PM_2.5_: Particulate matter 2.5
PTD: Preterm delivery
SO_2_: Sulfur dioxide

## References

[1] Walpole I, Zubrick S, Pontre J (1990). Is there a fetal effect with low to moderate alcohol use before or during pregnancy?. J Epidemiol Community Health.

[2] Simhan HN, Caritis SN (2007). Prevention of preterm delivery. N Engl J Med.

[3] Berkowitz GS, Papiernik E (1993). Epidemiology of preterm birth. Epidemiol Rev.

[4] Goldenberg RL, Rouse DJ (1998). Prevention of premature birth. N Engl J Med.

[5] Slattery MM, Morrison JJ (2002). Preterm delivery. Lancet.

[6] Cuevas KD (2005). The cost of prematurity: hospital charges at birth and frequency of rehospitalizations and acute care visits over the first year of life: a comparison by gestational age and birth weight. Am J Nurs.

[7] Petrou S (2005). The economic consequences of preterm birth during the first 10 years of life. BJOG.

[8] Petrou S (2003). The impact of preterm birth on hospital inpatient admissions and costs during the first 5 years of life. Pediatrics.

[9] Armstrong J (2007). 17 Progesterone for preterm birth prevention: a potential 2 billion dollar opportunity. Am J Obstet Gynecol.

[10] Li D, Liu L, Odouli R (2009). Presence of depressive symptoms during early pregnancy and the risk of preterm delivery: a prospective cohort study. Hum Reprod.

[11] Goldenberg RL (1996). Medical, psychosocial, and behavioral risk factors do not explain the increased risk for low birth weight among black women. Am J Obstet Gynecol.

[12] Fiscella K (1996). Race, perinatal outcome, and amniotic infection. Obstet Gynecol Surv.

[13] Knox IC, Hoerner JK (1950). The role of infection in premature rupture of the membranes. Am J Obstet Gynecol.

[14] Goldenberg RL, Hauth JC, Andrews WW (2000). Intrauterine infection and preterm delivery. N Engl J Med.

[15] Andres RL, Day MC (2000). Perinatal complications associated with maternal tobacco use. Semin Neonatol.

[16] Cnattingius S (2004). The epidemiology of smoking during pregnancy: smoking prevalence, maternal characteristics, and pregnancy outcomes. Nicotine Tob Res.

[17] Goldenberg RL (2008). Epidemiology and causes of preterm birth. Lancet.

[18] Wright EC (2014). Effect of elevated ambient temperature at parturition on duration of gestation, ruminal temperature, and endocrine function of fall-calving beef cows. J Anim Sci.

[19] Dreiling CE, Carman FS, Brown DE (1991). Maternal endocrine and fetal metabolic responses to heat stress. J Dairy Sci.

[20] De Rensis F, Scaramuzzi RJ (2003). Heat stress and seasonal effects on reproduction in the dairy cow--a review. Theriogenology.

[21] Wolfenson D (1993). Secretion of PGF2alpha and oxytocin during hyperthermia in cyclic and pregnant heifers. Theriogenology.

[22] Choi I (2015). Effects of prolonged exposure of mouse embryos to elevated temperatures on embryonic developmental competence. Reprod Biomed Online.

[23] Schifano P (2016). Heat and air pollution exposure as triggers of delivery: A survival analysis of population-based pregnancy cohorts in Rome and Barcelona. Environ Int.

[24] Schifano P (2013). Effect of ambient temperature and air pollutants on the risk of preterm birth, Rome 2001-2010. Environ Int.

[25] Lee SJ (2008). A time-series analysis of any short-term effects of meteorological and air pollution factors on preterm births in London, UK. Environ Res.

[26] Basu R, Malig B, Ostro B (2010). High ambient temperature and the risk of preterm delivery. Am J Epidemiol.

[27] Dadvand P (2011). Climate extremes and the length of gestation. Environ Health Perspect.

[28] Strand LB, Barnett AG, Tong S (2012). Maternal exposure to ambient temperature and the risks of preterm birth and stillbirth in Brisbane. Australia Am J Epidemiol.

[29] Auger N (2014). Extreme heat and risk of early delivery among preterm and term pregnancies. Epidemiology.

[30] Lajinian S (1997). An association between the heat-humidity index and preterm labor and delivery: a preliminary analysis. Am J Public Health.

[31] Yackerson N, Piura B, Sheiner E (2008). The influence of meteorological factors on the emergence of preterm delivery and preterm premature rupture of membrane. J Perinatol.

[32] Porter KR, Thomas SD, Whitman S (1999). The relation of gestation length to short-term heat stress. Am J Public Health.

[33] Gordon N (2012). A comparison of sociodemographic and health characteristics of the Kaiser Permanente Northern California membership derived from two data sources: The 2008 member health survey and the 2007 California health interview survey.

[34] Gordon N (2015). Similarity of the adult Kaiser Permanente Membership in Northern California to the Insured and General Population in Northern California: Statistics from the 2011-12 California Health Interview Survey.

[35] Maheswaran R (2016). Air pollution and subtypes, severity and vulnerability to ischemic stroke-a population based case-crossover study. PLoS One.

[36] Auger N (2016). Elevated outdoor temperatures and risk of stillbirth.

[37] Maclure M (1991). The case-crossover design: a method for studying transient effects on the risk of acute events. Am J Epidemiol.

[38] (CIMIS), C.I.M.S.

[39] National Climatic Data Center, C.R. 2012 September 23, 2015]; Available from: http://www.ncdc.noaa.gov/oa/climate/climateresources.html.

[40] Mart, A.Q.S.D. 2014.

[41] Stein CR (2009). Maternal ethnic ancestry and adverse perinatal outcomes in New York City. Am J Obstet Gynecol.

[42] Basu R, Feng WY, Ostro BD (2008). Characterizing temperature and mortality in nine California counties. Epidemiology.

[43] California Air Resources Board, A.Q.D.S., Planning and Technical Support Division. California Air Quality Data [Internet database]. 2006 August 18, 2008 [cited 2016 March 2, 2016]; Available from: http://www.arb.ca.gov/.

[44] DerSimonian R, Laird N (1986). Meta-analysis in clinical trials. Control Clin Trials.

[45] Frazier PA, Tix AP, Barron KE (2004). Testing moderator and mediator effects in counseling psychology research. J Couns Psychol.

[46] Strand LB, Barnett AG, Tong S (2011). Methodological challenges when estimating the effects of season and seasonal exposures on birth outcomes. BMC Med Res Methodol.

[47] Cox B (2016). Ambient temperature as a trigger of preterm delivery in a temperate climate.

[48] Wolf J, Armstrong B (2012). The association of season and temperature with adverse pregnancy outcome in two German states, a time-series analysis. PLoS One.

[49] Commission CE (2004). California Statewide Residential Appliance Saturation Study: Final Report.

[50] Karim SM (1968). Appearance of prostaglandin F2-alpha in human blood during labour. Br Med J.

[51] Kelly AJ, Tan B (2001). Intravenous oxytocin alone for cervical ripening and induction of labour. Cochrane Database Syst Rev.

[52] Kelly AJ (2009). Vaginal prostaglandin (PGE2 and PGF2a) for induction of labour at term. Cochrane Database Syst Rev.

[53] Lee SE (2008). Amniotic fluid prostaglandin concentrations increase before the onset of spontaneous labor at term. J Matern Fetal Neonatal Med.

[54] Satoh K (1979). Prostaglandin F2 alpha metabolite levels in plasma, amniotic fluid, and urine during pregnancy and labor. Am J Obstet Gynecol.

[55] Daugaard M, Rohde M, Jaattela M (2007). The heat shock protein 70 family: Highly homologous proteins with overlapping and distinct functions. FEBS Lett.

[56] Fukushima A (2005). Changes in serum levels of heat shock protein 70 in preterm delivery and pre-eclampsia. J Obstet Gynaecol Res.

[57] Hnat MD (2005). Heat shock protein-70 and 4-hydroxy-2-nonenal adducts in human placental villous tissue of normotensive, preeclamptic and intrauterine growth restricted pregnancies. Am J Obstet Gynecol.

[58] Peltier MR (2003). Immunology of term and preterm labor. Reprod Biol Endocrinol.

[59] Olson DM (2003). The role of prostaglandins in the initiation of parturition. Best Pract Res Clin Obstet Gynaecol.

[60] Challis JR (2002). Prostaglandins and mechanisms of preterm birth. Reproduction.

[61] Stan C (2002). Hydration for treatment of preterm labour. Cochrane Database Syst Rev.

[62] Bouchama A, Knochel JP (2002). Heat stroke. N Engl J Med.

[63] Astrand PO (2003). Textbook of Work Physiology: Physiological Bases of Exercise.

[64] Chen L (2010). Residential mobility during pregnancy and the potential for ambient air pollution exposure misclassification. Environ Res.

