# Supplementary material for: The impact of high apparent temperature on spontaneous preterm delivery: a case-crossover study
Source: Environ Health. 2017 Feb 1;16:5. doi: 10.1186/s12940-017-0209-5 (PMC5286689; doi:10.1186/s12940-017-0209-5)

Supplemental Figure 1. Map of Temperature Monitors in Northern California Counties Represented in the Study

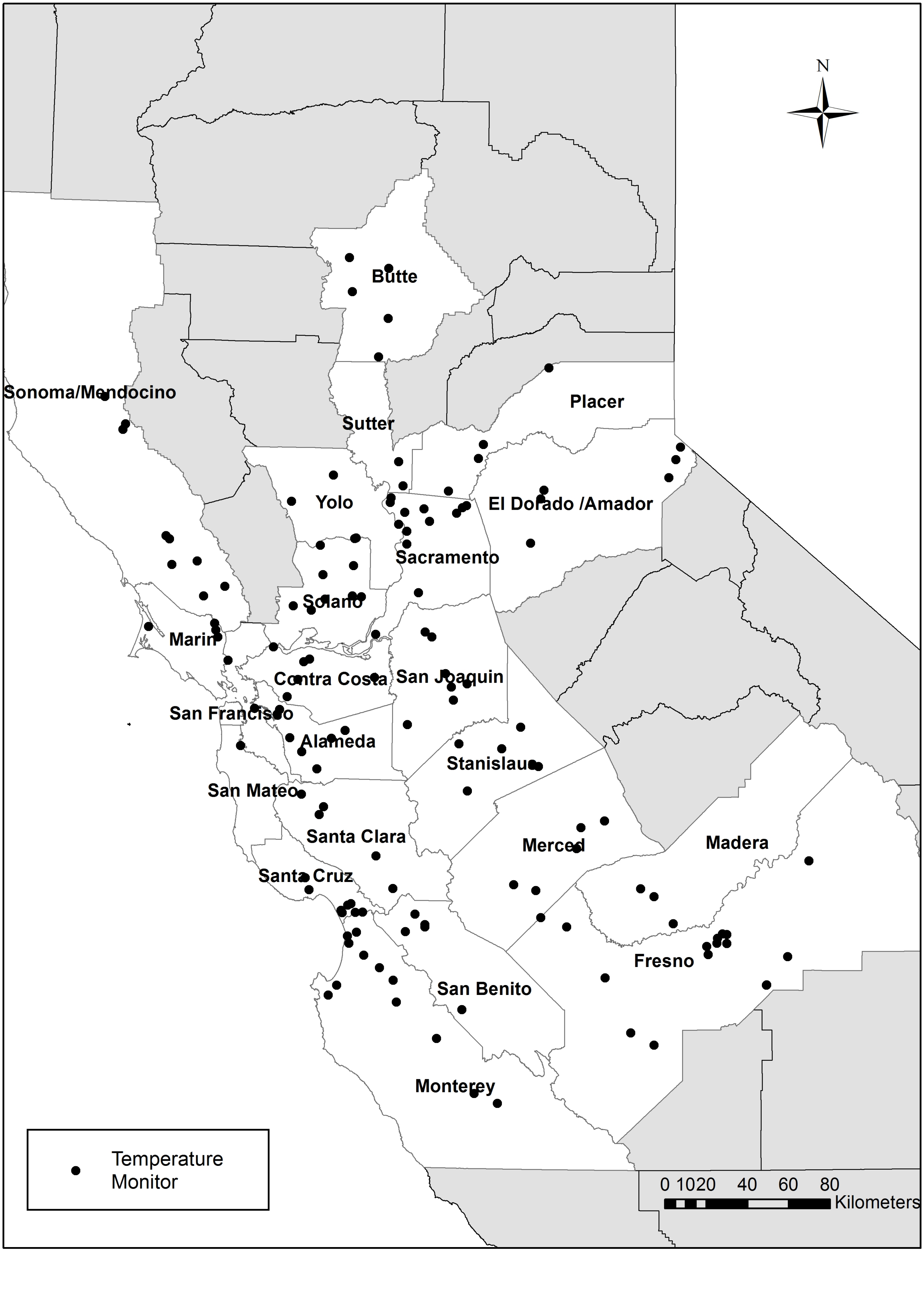

Supplement: Additional file 1: Figure S1. — Map of Temperature Monitors in Northern California Counties Represented in the Study. (PDF 2141 kb) [file 12940_2017_209_MOESM1_ESM.pdf]
